# Survival of Single-Unit Porcelain-Fused-to-Metal (PFM) and Metal Crowns Placed by Students at an Australian University Dental Clinic over a Five-Year Period

**DOI:** 10.3390/dj9060060

**Published:** 2021-05-28

**Authors:** Chris Carey, Nick Del Din, Jessica Lamb, Hazel Wright, Nigel D. Robb, Menaka Abuzar

**Affiliations:** 1 School of Medicine and Dentistry, Griffith University, Gold Coast 4215, Australia; chris.carey7@gmail.com (C.C.); nickdeldin@gmail.com (N.D.D.); jessica.lamb@live.com.au (J.L.); hazel.wright@hotmail.com (H.W.); n.robb@griffith.edu.au (N.D.R.); 2 Melbourne Dental School, University of Melbourne, Melbourne 3010, Australia

**Keywords:** crown, student, survival, complications, failures

## Abstract

The aim of this retrospective study was to determine the survival rate of single-unit porcelain-fused-to-metal (PFM) and metal crowns placed by dental students at an Australian university undergraduate dental clinic over a five-year period. Complications and the incidences of crown failures were recorded. Clinical records pertaining to single-unit PFM and metal crowns inserted over a five-year period were reviewed, including patient-related, tooth-related, and procedural factors for each crown. Crowns were evaluated as surviving, surviving with complications, or failed. Kaplan–Meier statistical analysis was used to estimate survival rate., This study is based on a sample of 232 (78.4%) PFM crowns and 64 (21.6%) metal crowns inserted between 2014 and 2018. Cumulatively, 224 (75.7%) were surviving, 48 (16.2%) were surviving but previously had complications, and 24 (8.1%) failed. The 5-year cumulative survival rate of all PFM and metal crowns was 83.9% (0.839 ± 0.038, Kaplan–Meier). The average survival time for all crowns was 4.432 ± 0.089 years. Comparatively, PFM crowns had a higher survival rate at 1 year (0.972 ± 0.010) and 2 years (0.919 ± 0.017), compared to metal crowns at 1 year (0.964 ± 0.011) and 2 years (0.894± 0.018). The survival rate of metal crowns remained constant from 2 years to 4 years and thereafter, whereas there was a continued decline in the survival rate of PFM crowns to 83.2% (0.832 ± 0.038) at 4 years and thereafter. Crowns placed on premolars had the highest cumulative survival rate whereas those placed on molars exhibited the lowest survival rate for the duration of the study period. Despite single-unit PFM crowns having a higher 1- and 2-year survival rate compared to metal crowns, metal crowns had a higher survival rate at 4 years and thereafter. Survival rates are comparable to previous studies.

## 1. Introduction

Porcelain-fused-to-metal (PFM) and metal crowns have been used to restore broken down teeth for the past three decades. Even though ceramic crowns have gained popularity due to its superior aesthetics and the ability to be produced through milling, the PFM and metal crowns are still the choice of treatment in many situations. Dental curricula in Australia are designed to provide undergraduates the knowledge and clinical skills required to provide PFM, metal, and ceramic crowns. Students commence learning fixed prosthodontics on mannikins in the pre-clinical simulation laboratory and enhance these skills by treating patients in the later years of the program. The students are closely supervised in the clinics by experienced teachers, and it is expected that these crowns remain functional and survive intra orally for an acceptable length of time.

Few studies have evaluated the survival rates of indirect restorations placed by dental students. Those that have, considered restorations placed over 30 years ago [1], were conducted outside Australia [1,2], are related to restorations other than single-unit crowns [3,4] (including provisional restorations [2]) and evaluated materials other than PFM and metal [5,6]. This is the first study to consider the survival of indirect single-unit PFM and metal crowns placed by dental students at an Australian dental school.

A retrospective study conducted into PFM crowns placed by dental students at the University of Oulu in Finland reported a 78% survival rate after 20 years follow-up [1]. Multiple studies have reported survival and complication rates of PFM and metal crowns placed by dentists in private practices and the public sector. Studies identified a 3- to 5-year survival rate of between 92% and 97.6% for PFM crowns [7,8,9,10]. One study found that metal crowns had a 5-year survival rate of 80% [11].

Complications affect the survival of crowns. Studies have considered a number of complications broadly categorised as biological, patient-related and technical [8]. A systematic review conducted by Patel et al. [12] into clinical studies of fixed prostheses reported the frequency of studies that identified various complications. Among the most common complications reported were biological complications, caries, porcelain fracture and mechanical complications. Other reported complications that affect the survival of crowns placed by dentists include endodontic complications [10,11], secondary caries [7,10], chipping and porcelain fracture [7,10,11], tooth fracture [10], and periodontitis [7]. 

Based on existing literature, a classification system was developed to evaluate whether a crown was ‘surviving’, ‘surviving with complications’ or had ‘failed’. A surviving crown was defined in some studies as a crown still in place after a given period of time, regardless of whether modification was required to the crown or not [8,13]. In contrast, a crown failed if it required replacement [14], or extraction of the tooth [9], or it had been lost [15]. 

The aim of this study was to investigate the survival rate of both single-unit PFM and metal crowns placed by dental students at an Australian university undergraduate dental clinic over a five-year period, as well as analysing the number and type of complications and failures. The study also intended to identify the incidences and reasons for crown failures.

## 2. Materials and Methods

A retrospective cohort study design was implemented. Historical searches of relevant Australian Dental Association (ADA) treatment codes (Table 1) within an Australian university’s patient management system, Titanium (Titanium Software Inc. Houston, TX, USA) were conducted. The inclusion criteria were single-unit PFM or metal crowns placed by dental students in the undergraduate clinic between January 2014 and December 2018. Ethical approval was granted by the university to access patient records. 

**Figure 1:**
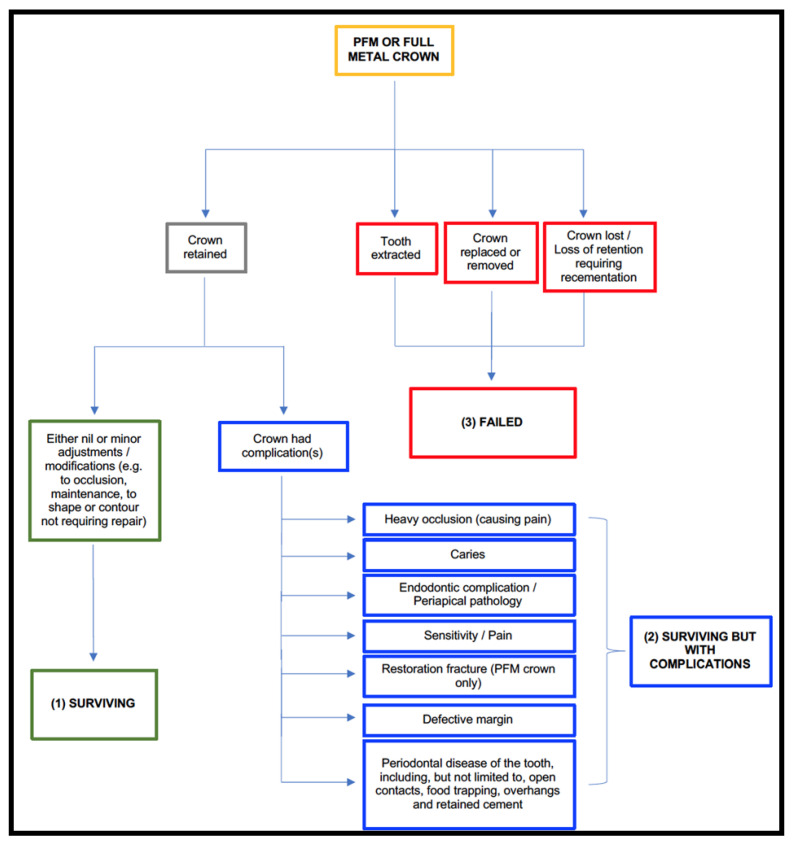
Classification flow chart.

Crowns were excluded if a patient had not attended an examination within three years of crown placement or if the crown was not placed at the student dental clinic. Crowns that were ‘surviving’ were defined as crowns that were retained in position, despite minor adjustments or modifications to occlusion, shape or contour and without complications [8,15]. Crowns ‘surviving with complications’ were defined as crowns that were retained in position but had previous complications including pain, heavy occlusion causing pain, caries, endodontic pathology, fracture, defective margin or periodontal disease [13]. Crowns that had ‘failed’ were those that had been lost and required re-cementation, or had been replaced [14], or the tooth had been extracted [9]. Figure 1 sets out a flow chart used during the analysis phase to assist with the categorisation of crown.

Preliminary search of Titanium yielded 1380 crowns. Four final year masters-level dental students each reviewed one-quarter of the preliminary search results. Three hundred crowns met the inclusion criteria. Patient records in Titanium were reviewed for 296 crowns to obtain relevant data, including the patient’s age at time of crown placement, gender, date of placement and latest examination, tooth number, treatment history of the relevant tooth, year level of the operator, the material and cement used, and, if appropriate, the date a complication arose. An assessment was made using the available data, Classification Flow Chart and patient treatment notes to classify the crown status as either: (i) surviving; (ii) surviving with complications; or (iii) failed.

Calibration of data analysers was achieved by randomly and independently cross-checking the preliminary search results allocated to each examiner. Calibration occurred by one examiner reviewing approximately 7% of the allocated search results given to another examiner who reciprocated the review process. Each examiner evaluated the search results for inclusion and exclusion criteria. After independently reviewing the search results, examiners met to discuss discrepancies and finalised the Classification Flow Chart. Verification of data entry took place after completing the review of the preliminary search results. Examiners collectively checked accuracy of data entry, interpretation of patient notes, and assessment of crown status for 15% of the crowns meeting the inclusion criteria.

### Statistical Analysis

Categorical variables are presented as absolute numbers and percentages. The mean and standard deviation of continuous variables that are normally distributed are also provided. 

The survival rate of PFM and metal crowns was calculated using Kaplan–Meier statistical analysis to determine the probability estimate of survival [16]. The Kaplan–Meier method is a nonparametric method used to estimate the probability of survival past given time points. This method also allows comparison of survival distributions of two or more attributes or factors for equality. The input variables used for survival analysis are: (1) ‘Time’ which is the duration from the start until an event occurs or when the data becomes censored. In this study time has been measured in months; (2) ‘Status’ which includes events of ‘failures’ and participant dropouts; and (3) ‘Intervention’ which includes between-participants ‘factor’. In this study, a survival comparison has been made based on crown position (anterior teeth, premolars and molars) and crown material (PFM and metal). To determine the significance of differences between survival distributions, the following tests have been carried out: Log Rank (Mantel-Cox) Test, Breslow (Generalised Wilcoxon) Test, and Tarone–Ware Test.

Survival of PFM and metal crowns for the purposes of Kaplan–Meier analysis was deemed as a crown in situ at the time of the most recent examination unless the crown was recemented in which case the crown was deemed to have failed. There were 272 crowns that were surviving or surviving but previously had complications and so were in situ at the most recent examination. These were considered as surviving for the purposes of the Kaplan–Meier analysis, while 24 crowns had failed. In addition to cumulative survival rate, average survival time for crowns was also calculated. Statistical Package for the Social Sciences version 24.0 (SPSS, IBM) was used to analyse the dataset and conduct Kaplan–Meier survival rate calculations.

## 3. Results

Over the five-year period, 296 PFM and metal single-unit crowns that were placed in the undergraduate clinic by dental students in 234 patients of which 157 (52.7%) were male and 141 (47.3%) were female. Some patients had multiple crowns placed. The mean age of the patients was 60.71 years (SD = 12.58). 

For those crowns placed over the five-year period, 224 (75.7%) were surviving, 48 (16.2%) were surviving but previously had complications, and 24 (8.1%) had failed. Over 90% of crowns were placed by students in their final year of the dental programme. Of the 296 crowns, 232 (78.4%) were PFM crowns and 64 (21.6%) were metal crowns (Table 2). 

Forty-eight crowns (16.1%) were surviving but previously had complications (Table 3). Pain was the highest reported complication (n = 23), followed by periodontal disease associated with the crowned tooth (n = 21). Caries was associated with only one crown (0.3%). The mean interval between cementation and the complication arising was 436.31 days (SD = 456.6). 

Most failed crowns had to be replaced or removed (n = 14). Of the failed crowns, 19 PFM crowns failed compared to 5 metal crowns (Table 4). Seven crowns failed due to extraction of the related tooth. Three crowns required re-cementation and were each recemented on one occasion. No crowns were lost. 

### Survival Analysis

This retrospective study is based on the data collection over five years, from January 2014 till December 2018. However, the time of the last event was the 59th month, which is just short of five years (4.917 years). However, for interpretation purposes for crown survival, we have used the expression ‘5 years’ to indicate the extent of the study period. 

**Figure 2:**
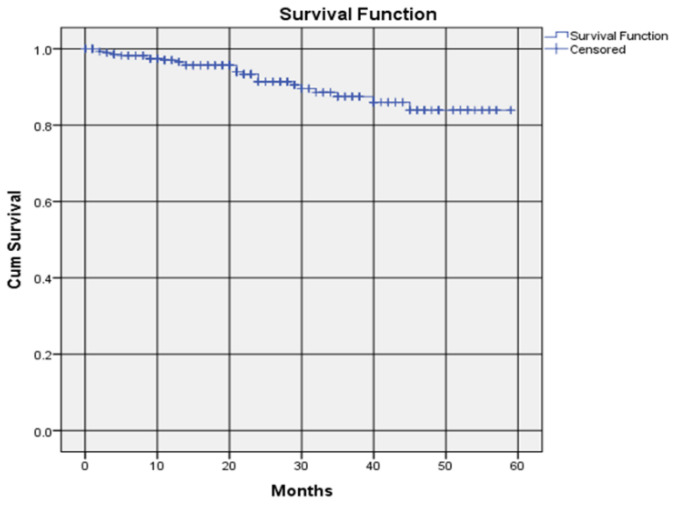
Survival rate of all PFM and metal crowns placed by students.

PFM and metal crowns together had a survival rate of 97.0%, i.e., 0.970 ± 0.011, (Kaplan–Meier Estimate ± SE) at year one, 91.4% (0.914 ± 0.017, Kaplan–Meier) at year two, 87.5% (0.875 ± 0.027, Kaplan–Meier) at year three and 83.9% survival rate (0.839 ± 0.038, Kaplan–Meier) at year four and beyond (Figure 2). The average survival time was 4.432 ± 0.089 (Mean ± SE) years.

**Figure 3:**
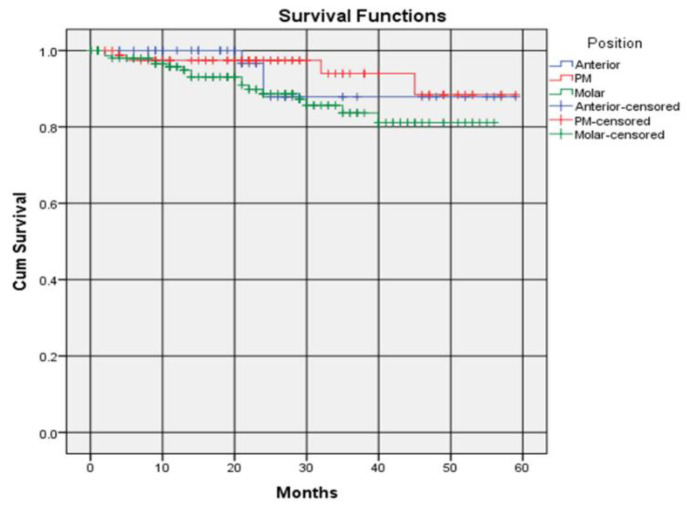
Survival rate of crowns according to position.

The survival rate of crowns differed slightly according to whether the crowns were placed on anterior teeth, premolars or molars. Crowns placed on premolars had the highest 5-year survival rate of 88.4% (0.884 ± 0.073, Kaplan–Meier), compared to 87.9% (0.879 ± 0.061, Kaplan–Meier) for anterior teeth and 81.8% (0.818 ± 0.047, Kaplan–Meier) for molars. The survival rate of crowns placed on anterior teeth remained constant at year 2 and afterwards. The survival rate of crowns placed on premolars and molars remained constant after 4 years. (Figure 3). There were no significant differences in-between the survival distributions of the three positions of the crown placements (Table 5). The average survival times were 4.651 ± 0.179, 4.586 ± 0.140 and 4.084 ± 0.122 years for the anterior, premolar and molar regions, respectively. 

**Figure 4:**
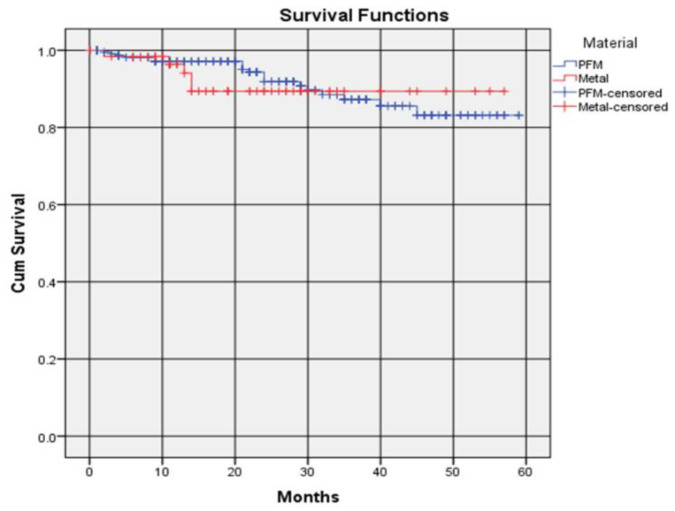
Survival rate of crowns according to material.

PFM crowns recorded a higher 1-year survival rate compared to metal crowns, 97.2% (0.972 ± 0.010, Kaplan–Meier) and 96.4% (0.964 ± 0.011, Kaplan–Meier), respectively (Figure 4). The survival rate of PFM crowns was again higher than metal crowns at 2 years, with a survival rate of 91.9% (0.919 ± 0.017, Kaplan–Meier) compared to 89.4% (0.894 ± 0.018, Kaplan–Meier) for metal crowns. The survival rate of metal crowns up to 3-year was higher than PFM crowns as the survival rate of PFM crowns continued to decrease to 83.2% (0.832 ± 0.038, Kaplan–Meier) at 4 years and remained unchanged thereafter. The survival rate of metal crowns was the same between 2 and 4 years. There were no significant differences between the survival distributions of the two crown materials (Table 6). The average survival times were 4.446 ± 0.179 and 4.276 ± 0.182 for PFM and metal, respectively.

## 4. Discussion

This study found that the survival rate of single-unit PFM crowns was 83.2% over 5 years. The 5-year survival rate of single-unit metal crowns was 89.4%. Prior to this study, the survival rate of single-unit PFM and metal crowns placed by dental students in Australia had not been investigated. 

A retrospective study conducted by Näpänkangas and Raustia [1] at the University of Oulu, Finland, considered the survival rate of PFM crowns placed between 1984 and 1987 by dental students. The study involved 50 patients who each attended a follow-up clinical examination to determine the 20-year survival rate. The 20-year survival rate of those PFM crowns was 78%, where the ‘survived’ crowns were in situ at the time of the follow-up examination regardless of whether those crowns had been recemented or experienced porcelain fractures [1]. 

In our study, the 5-year survival rate of PFM crowns was 5.2% higher than the 20-year survival rate in the study by Näpänkangas and Raustia [1], which included no specific data with respect to 5-year outcomes to compare. Our study defined survival more narrowly by deeming crowns that had been recemented as failures. This could have led to a lower survival rate in our study than if the definition of survival been construed more broadly. In the Finnish study [1], however, a specialist clinician performed follow-up clinical and radiographic examinations to determine the crown status of 100 PFM crowns. While our study considered a larger number of PFM crowns (N = 232), it relied on information recorded by dental students in patient files based on examinations conducted of patients. The authors cannot guarantee the quality of those examinations conducted. It was accepted that patient notes were accurate and correct ADA item codes charted. Detailed patient-specific variables, including parafunctional habits, caries risk and medical history, were not considered. 

Several studies have considered the survival rate of PFM crowns placed by dentists in private practice or public dental clinics across various time periods. Overmeer et al. [8] considered PFM and composite single crowns placed within the public dental service in Sweden. They reported a 5-year survival rate of PFM crowns of 93% using logistic regression analysis. Overmeer et al. [8] defined survival to be a crown in place at the 5-year follow-up regardless of whether modifications had been performed. They employed the same method used in our study to determine crown status by evaluating patient clinical records without independent clinical examination by the authors. Overmeer et al. [8] did, however, consider available radiographs up to the time of data collection.

Behr et al. [7] conducted a study including 997 PFM crowns placed between 1984 and 2009. They found a 5-year survival rate of 96.4% for PFM crowns placed anteriorly, and 97.5% for those crowns placed posteriorly. Our study further divided posterior crowns into premolars and molars. The 5-year survival rate of crowns placed on anterior teeth was slightly lower, with an 87.9% survival rate. Our study also reported a survival rate of 88.4% for premolars and 81.1% for molars, compared with the survival rate for posteriorly placed crowns. Notably, the survival rate was higher for anteriorly placed crowns in our study, compared to posteriorly placed crowns.

A study by Rinke et al. [9] into PFM crowns placed in private practice over 3 years reported a survival rate of 97.6%, which compares to the 3-year survival rate of 87.2% for PFM crowns placed as part of our study. Again, the comparison study’s definition of survival was broader as only crowns that had suffered an unacceptable fracture, were replaced or required extraction of the associated tooth were deemed to have failed.

Previous research conducted by Burke and Lucarotti [11] into the survival rates of, inter alia, metal crowns, placed by dentists within the public system in England and Wales reported a lower 5-year survival rate of 80% compared to the 89.4% survival rate reported in our study. The study by Burke and Lucarotti [11] was more extensive as it considered 7817 metal crowns placed over an 11-year period.

In our study, 23 patients reported pain associated with their crown, of which 17 were related to PFM crowns (7.3% of all PFM crowns) and 6 related to metal crowns (9.4% of all metal crowns). In the study by Näpänkangas and Raustia [1], 11% of patients with PFM crowns experienced pain over a 20-year period. Periodontal disease was the second highest reported complication in our study (N = 21) of which 13 were related to PFM crowns (5.6% of all PFM crowns) and 8 metal crowns (12.5% of all metal crowns). This can be compared with 14.4% of PFM crowns affected by periodontal disease in the study by Behr et al. [7] where clinical data of crowns inserted from January 1984 to May 2009 was investigated. Our study also reported lower incidences of caries in PFM crowns, 0.4% compared to 1.3% reported in Behr et al. [7].

A study by Raedel et al. [17] evaluated the longevity of crowns by analysing a massive data warehouse (N = 192,868) of a major German national health insurance company (BARMER, Berlin, Germany) over six years. The cumulative 5-year survival rate of ‘metal’ and ‘metal with ceramic crowns’ (veneered on the vestibular aspects) which included only anterior teeth and premolars was 90.4%. The survival rates of anterior crowns (87.9%) and premolars (88.4%) found in our study are comparable. The slightly lower survival rate in our study may be due to the difference in the sample size and clinical context (e.g., qualified clinicians vs. student clinicians).

The Kaplan–Meier method to calculate the survival rate of PFM crowns and metal crowns provides a statistical prediction of the survival rate rather than the actual observed data [16]. The survival rate is determined by the definitions of survival and failure used. Some authors recognised that the survival and failure rates may misrepresent the actual position given that some patients may have a crown in place that requires removal but would be considered to be surviving based on its presence [18]. Other patients may have had a crown removed for aesthetic reasons despite no functional failure of the restoration [7].

Due to the design of this retrospective study, there could have been some selection bias. Some patients meeting the inclusion criteria attended an examination within three years of crown cementation but did not return to the clinic for further treatment. The status of those crowns was assumed to be as at the examination date, which may overrepresent the survival rate given the crown may have subsequently failed. It is also possible that dissatisfied patients may not return to the clinic [4]. Equally, many patients did not return to the clinic for an examination following crown placement. These crowns were omitted from the study. 

This study provides a foundation for future research into the survival rates of crowns placed by students at other dental schools in order to compare the survival rates recorded in this study with a view to reducing the number of complications and failures, develop guidelines for crown placement and improve patient outcomes [2]. Further studies could also be conducted to incorporate a clinical examination component using trained examiners at various follow-up intervals to determine crown status. The survival rate of other crown materials (e.g., Zirconia, lithium disilicate), as well as fixed prostheses with more than one unit, would also be valuable in circumstances where these restorations are placed in a university setting. 

## 5. Conclusions

The 5-year cumulative survival rate of all crowns placed by students in a university dental clinic was 83.9% (0.839 ± 0.038, Kaplan–Meier) and the average survival time was 4.432 ± 0.089 years. The survival distributions of crowns differed slightly for the two materials (PFM and metal) though the differences were not statistically significant (*p* > 0.05). The average survival times of crowns were 4.446 ± 0.179 and 4.276 ± 0.182 years for PFM and metal, respectively. The survival distributions also differed based on crown placements (anterior tooth, premolar, molar) though the differences were not significant (*p* > 0.05). The average survival times were 4.651 ± 0.179, 4.586± 0.140 and 4.084 ± 0.122 years for the anterior, premolar and molar positions, respectively. Complications experienced by crowns remaining in situ included pain, caries, heavy occlusion, endodontic/periapical pathology, restoration fracture, defective margin and periodontal disease. Most failed crowns required replacement or removal.

## Figures and Tables

**Table 1:** ADA codes charted as part of patient treatment.

ADA Code	Description
595	Removal of indirect restoration
596	Recementing of indirect restoration
615	Full crown—veneered—indirect
618	Full crown—metallic—indirect
651	Recementing crown or veneer
658	Repair of crown, bridge or splint—indirect
689	Repair of crown, bridge or splint—direct

Note: ‘N’ and ‘R’ codes were also reviewed indicating a no charge item or retreatment.

**Table 2:** Summary information of crowns (N = 296).

Variables		PFM Crown N (%)	Metal Crown N (%)
Position			
	Anterior	54 (23.3)	0 (0.0)
	Premolar	80 (34.5)	5 (7.8)
	Molar	98 (42.2)	59 (92.2)
Vitality			
	Vital	100 (43.7)	32 (50.0)
	Non-Vital	129 (56.3)	32 (50.0)
Root-canal treated			
	Yes	127 (55.9)	29 (46.8)
	No	100 (44.1)	33 (53.2)
Post and Core			
	Yes	61 (26.5)	10 (15.6)
	No	169 (73.5)	54 (84.4)
Operator			
	4th Year student	22 (9.5)	2 (3.1)
	5th Year student	210 (90.5)	62 (96.9)
Cement			
	Resin modified glass ionomer	211 (92.5)	62 (96.9)
	Glass ionomer	2 (0.9)	0 (0.0)
	Resin	13 (5.7)	2 (3.1)
	Temp Bond NE	2 (0.9)	0 (0.0)
Crown status			
	Surviving	178 (76.7)	46 (71.9)
	Surviving with complications	35 (15.1)	13 (20.3)
	Failed	19 (8.2)	5 (7.8)

**Table 3:** Complications associated with PFM and metal crowns (N = 296).

Complication		PFM Crown N (%)	Metal Crown N (%)
Pain			
	Yes	17 (7.3)	6 (9.4)
	No	215 (92.7)	58 (90.6)
Caries			
	Yes	1 (0.4)	0 (0.0)
	No	231 (99.6)	64 (100.0)
Heavy occlusion			
	Yes	8 (3.4)	4 (6.3)
	No	224 (96.6)	60 (93.8)
Endodontic/Periapical pathology			
	Yes	5 (2.2)	1 (1.6)
	No	227 (97.8)	63 (98.4)
Restoration fracture			
	Yes	2 (0.9)	0 (0.0)
	No	230 (99.1)	64 (100.0)
Defective margin			
	Yes	6 (2.6)	2 (3.1)
	No	226 (97.4)	62 (96.9)
Periodontal disease			
	Yes	13 (5.6)	8 (12.5)
	No	219 (94.4)	56 (87.5)

**Table 4:** Reasons for failure of PFM and metal crowns (N = 24).

Reason for Failure	PFM Crown N (%)	Metal Crown N (%)
Tooth extracted	6 (31.6)	1 (20.0)
Crown replaced or removed	11 (57.9)	3 (60.0)
Crown lost or recemented	2 (10.5)	1 (20.0)

**Table 5:** Test of survival distributions for three different crown positions.

Test	χ^2^	*p*
Log Rank (Mantel–Cox)	4.571	0.102
Breslow (Generalised Wilcoxon)	5.677	0.059
Tarone–Ware	5.502	0.064

**Table 6:** Test of survival distributions for two different crown materials.

Test	χ^2^	*p*
Log Rank (Mantel–Cox)	0.452	0.502
Breslow (Generalised Wilcoxon)	1.544	0.214
Tarone–Ware	1.071	0.301

## Data Availability

Data sharing for this paper is not permissible due to the restrictions placed on patients’ records.

## References

[1] Näpänkangas R., Raustia A. (2008). Twenty-year follow-up of metal-ceramic single crowns: A retrospective study. Int. J. Prosthodont..

[2] Hyde J.D., Bader J.A., Shugars D.A. (2007). Provisional crown failures in dental school predoctoral clinics. J. Dent. Educ..

[3] Hochman N., Mitelman L., Hadani P.E., Zalkind M. (2003). A clinical and radiographic evaluation of fixed partial dentures (FPDs) prepared by dental school students: A retrospective study. J. Oral Rehabil..

[4] Bühler J., Naef M.A., Amato M., Krastl G., Weiger R., Zitzmann N.U. (2017). Partial ceramic crowns prepared by dental students: Clinical performance up to five years. J. Dent. Educ..

[5] Tartaglia G.M., Sidoti E., Sforza C. (2011). A 3-year follow-up study of all-ceramic single and multiple crowns performed in a private practice: A prospective case series. Clinics.

[6] Näpänkangas R., Pihlaja J., Raustia A. (2015). Outcome of zirconia single crowns made by predoctoral dental students: A clinical retrospective study after 2 to 6 years of clinical service. J. Prosthet. Dent..

[7] Behr M., Zeman F., Baitinger T., Galler J., Koller M., Handel G., Rosentritt M. (2014). The clinical performance of porcelain-fused-to-metal precious alloy single crowns: Chipping, recurrent caries, periodontitis, and loss of retention. Int. J. Prosthodont..

[8] Overmeer J., Narby B., Hjalmarsson L., Arnrup K., Eliasson A. (2016). A retrospective multicenter study comparing metal-ceramic and composite single crowns performed in public general dentistry: 5-year results. Acta Biomater. Odontol. Scand..

[9] Rinke S., Schäfer S., Lange K., Gersdorff N., Roediger M. (2013). Practice-based clinical evaluation of metal–ceramic and zirconia molar crowns: 3-year results. J. Oral Rehabil..

[10] Heintze S.D., Rousson V. (2010). Survival of zirconia- and metal-supported fixed dental prostheses: A systematic review. Int. J. Prosthodont..

[11] Burke F.J.T., Lucarotti P.S.K. (2009). Ten-year outcome of crowns placed within the general dental services in England and Wales. J. Dent..

[12] Patel D.R., O’Brien T., Petrie A., Petridis H. (2014). A systematic review of outcome measurements and quality of studies evaluating fixed tooth-supported restorations. J. Prosthodont..

[13] Pjetursson B.E., Valente N.A., Strasding M., Zwahlen M., Liu S., Sailer I. (2018). A systematic review of the survival and complication rates of zirconia-ceramic and metal-ceramic single crowns. Clin. Oral Implants Res..

[14] Anusavice K.J. (2012). Standardizing failure, success, and survival decisions in clinical studies of ceramic and metal–ceramic fixed dental prostheses. Dent. Mater..

[15] Olley R.C., Andiappan M., Frost P.M. (2018). An up to 50-year follow-up of crown and veneer survival in a dental practice. J. Prosthet. Dent..

[16] Kaplan E.L., Meier P. (1958). Nonparametric estimation from incomplete observations. J. Am. Stat. Assoc..

[17] Raedel M., Priess H.-W., Bohm S., Walter M.H. (2020). Six-year survival of single crowns A massive data analysis. J. Dent..

[18] Scurria M.S., Bader J.D., Shugars D.A. (1998). Meta-analysis of fixed partial denture survival: Prostheses and abutments. J. Prosthet. Dent..

